# Eye Tracking in Driver Attention Research—How Gaze Data Interpretations Influence What We Learn

**DOI:** 10.3389/fnrgo.2021.778043

**Published:** 2021-12-08

**Authors:** Christer Ahlström, Katja Kircher, Marcus Nyström, Benjamin Wolfe

**Affiliations:** ^1^Swedish National Road and Transport Research Institute (VTI), Linköping, Sweden; ^2^Department of Biomedical Engineering, Linköping University, Linköping, Sweden; ^3^Lund University Humanities Lab, Lund, Sweden; ^4^Department of Psychology, University of Toronto Mississauga, Mississauga, ON, Canada

**Keywords:** eye tracking (ET), driving (veh), distraction and inattention, purpose-based analysis, coding scheme, context, relevance

## Abstract

Eye tracking (ET) has been used extensively in driver attention research. Amongst other findings, ET data have increased our knowledge about what drivers look at in different traffic environments and how they distribute their glances when interacting with non-driving related tasks. Eye tracking is also the go-to method when determining driver distraction via glance target classification. At the same time, eye trackers are limited in the sense that they can only objectively measure the gaze direction. To learn more about why drivers look where they do, what information they acquire foveally and peripherally, how the road environment and traffic situation affect their behavior, and how their own expertise influences their actions, it is necessary to go beyond counting the targets that the driver foveates. In this perspective paper, we suggest a glance analysis approach that classifies glances based on their *purpose*. The main idea is to consider not only the intention behind each glance, but to also account for what is relevant in the surrounding scene, regardless of whether the driver has looked there or not. In essence, the old approaches, unaware as they are of the larger context or motivation behind eye movements, have taken us as far as they can. We propose this more integrative approach to gain a better understanding of the complexity of drivers' informational needs and how they satisfy them in the moment.

## Introduction

A video with an overlaid fixation cross that shows where the driver's gaze is focused relative to the scenery is a powerful visualization. From such data, it is possible to derive objective and quantitative results like gaze direction, dwell time, and glance frequency to objects and locations. In driver attention research, eye movement analysis has been used to learn more about gaze behavior associated with mobile phone use (Tivesten and Dozza, 2014), the distribution of eyes-off-road durations (Liang et al., 2012), where drivers look at the road to maintain a smooth travel path (Lappi et al., 2013), where drivers sample visual information when driving through intersections (Kircher and Ahlström, 2020), etc.

Despite everything that eye movement analysis has taught us about driver behavior, one should be aware of some fundamental limitations in using eye tracking (ET) to study driver attention and behavior. *First*, eye trackers only measure where and for how long we look in a certain direction or at a certain target. It is not a direct overt measure of visual attention (e.g., Deubel and Schneider, 1996), and information about the purpose of the glance or what information the brain cognitively processes during the glance can be very difficult to access (cf. Viviani, 1990). *Second*, there is no method to directly measure information acquisition via peripheral vision that works in real-world applications, even though research indicates that drivers are aware of much more than what is being foveated (Underwood et al., 2003). Wolfe et al. (2020) even argue that peripheral input provides much of the information the driver needs, both at a global level (the gist of the scene, acquired in parallel) and at a local level (providing information to guide search processes and eye movements more generally). *Third*, it has been shown that not all foveated information is processed (Simons, 2000; Mack, 2003). This is often referred to as looked but failed to see or inattentional blindness. *Finally*, eye movement data do not provide an easy way to determine whether the sampled information was relevant, necessary, and sufficient for the driver in the current situation (Kircher and Ahlström, 2018; Wolfe et al., 2020). Considering these limitations, it is clear that driver attention assessments cannot be based on single foveations, without also considering glance history and the present traffic situation.

An alternative to interpreting a driver's visual information sampling gaze by gaze, target by target, is to consider visual information acquisition in driving as a task where many different glance strategies can be equally appropriate. The basic idea is that an attentive driver has a “good enough” mental representation of the current situation, containing imperfect but adequate information about the surrounding scene (Summala, 2007; Hancock et al., 2009). As suggested by Wolfe et al. (2020), this mental representation is built from information acquired via a series of context-guided glances in combination with peripheral vision, using data from the attentive and pre-attentive stages of information acquisition, and possibly from other sources. The representation can only be sufficient if enough relevant information is included. We would need to know where and at what drivers look and for what reason (including what they see with peripheral vision), their intended travel path and other tasks they are doing, and preferably also their familiarity and experience with the given situation. The dilemma is that even with accurate ET, co-registered with a recording of the driver's environment, and an experimental design that controls for travel path and tasks, we still would not be able to measure (i) information sampled via peripheral vision and (ii) the top-down processes that are known to influence why and from where information is sampled (Kircher and Ahlström, 2018). Note that from a driver attention perspective, it is not even enough to investigate if the sampled information is relevant and if it has been sampled sufficiently, it is also necessary to check that no relevant information was missed. Still, when combined with additional data and an innovative data reduction approach, gaze data can still be an asset for monitoring driver attention.

In this perspective paper, we compare different approaches to encode and interpret ET data that has been used in the field of driver attention research. For each approach we discuss the data needed, the implicit or explicit definition of an attentive driver, the typical results that can be obtained, and the conclusions that are likely to be drawn (summarized in Table 1). In addition to classifying gaze data based on *direction* and on the foveated *target*, we also include an approach that classifies glances based on their *purpose*. In this paper, we argue that the purpose-based approach provides added value for understanding context-based driver attention.

**Table 1:** Methodological aspects to consider when applying direction, target, and purpose-based approaches to eye tracking data.

**Approach**	**Direction**	**Target**	**Purpose**
**Strategy**	Identify the glance direction	Identify the foveated glance target	Identify the probable reason for the glance
**Actual coding**	To the right OR away from forward	Bicyclist	Checking for relevant traffic from right
**External knowledge needed for classification**	Coordinate system determining forward	View of outside world	View of outside world, traffic rules that apply, intended direction of travel
**Coding method**	Real-time automated coding is available	Manual or semi-automated	Manual
**Typical result**	Frequency and duration of eyes-off-road	Frequency and duration of glances toward (type of) target or area	Frequency and duration (or neglection) of target or area in context of relevance
**Typical research questions**	How much do drivers look in certain directions or away from the forward roadway?	How much do drivers look at various targets?	How often are relevant areas or targets neglected?

## Direction-, Target-, and Purpose-Based Eye Movement Interpretations

**Figure 1:**
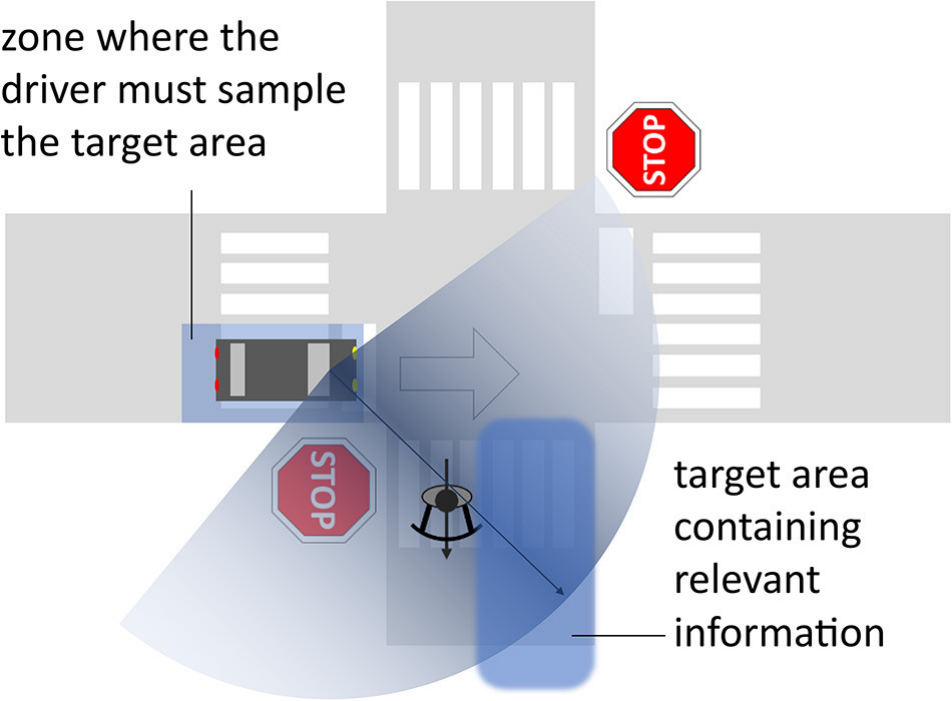
Illustrative example showing a driver who is approaching an intersection with the intention to drive straight ahead. A bicyclist is leaving the intersection. While in the zone with a good view of the intersection, before entering it, the driver is looking right (thin arrow—foveal vision, shaded sector—peripheral vision), checking for traffic potentially present in the target area. A similar check for traffic from the left is required, too (but not illustrated in the figure).

To understand the differences between the *direction-, target-*, and *purpose-based* approaches when studying driver attention with ET, we start with the illustration in Figure 1. A driver intending to continue straight ahead is approaching an intersection. At the same time, a bicyclist is leaving the intersection on the main road. The driver glances to the right, foveating the bicyclist.

### Direction-Based Approach

In the direction-based approach, the gaze direction is registered, typically as forward, up, down, left, and right. This approach is typically used when the eye movements are recorded in a coordinate system that is fixed relative to a vehicle-mounted remote eye tracker. It is then easy to extract the gaze direction, without the need for a scene camera. In Figure 1, the direction-based approach would register a glance to the right.

The direction-based approach is often used to compute indicators like “eyes off road” or “percent road center” (PRC; Victor et al., 2005) and it can be employed in real-time with automated data encoding. A driver is considered attentive when directing a minimum percentage of glances within a sliding window to the “road center,” which would be the relevant area. A drawback with this approach is that the relevant area is typically defined as “forward,” regardless of where relevant information is positioned relative to the car. Data fusion makes it possible to define more elaborate relevant areas that can be coupled to the direction of the gaze. For example, if the eye tracker data are associated with a world model of the vehicle's cockpit, glances to relevant areas representing the speedometer, and the mirrors can be treated differently than other off-road glances (Ahlstrom et al., 2013). To some extent, situational circumstances can also be integrated via map data and proximity sensors, allowing automated adjustments of the relevant area(s), for example by taking road curvature (Ahlstrom et al., 2011) and intersections (Ahlström et al., 2021) into account. Data fusion with other data sources is still uncommon, and eye movements are to a large extent interpreted without situational information in the direction-based approach. It is thus unknown *what* the driver glances at and *why*.

### Target-Based Approach

The *what*-question is typically answered by manual coding of scene videos with a gaze overlay, either from a remote or a head-mounted eye tracker. Solutions based on deep learning are also emerging where it is possible to automatically recognize objects in the videos and denote when the point-of-gaze intersects these objects (Panetta et al., 2020). To distinguish targets with similar XY-coordinates it may also be possible to use depth information from binocular eye trackers.

Glance *targets* are coded according to the target type, such as “bicyclist,” “traffic sign,” or “mobile phone” (Kircher and Ahlström, 2020). The glance to the right in Figure 1 would be coded as “bicyclist.” Target types that have a connection to traffic are often tacitly assumed to be relevant for driving, regardless of whether they contribute any relevant information in the current situation or not, like the bicyclist in Figure 1 who is not relevant considering the driver's upcoming travel path. *Direction-* and *target-based* approaches commonly infer driver distraction when glances are directed away from forward or toward target types that are deemed irrelevant for driving (Halin et al., 2021). While widely accepted, these approaches often miss the important aspects of context, if relevant information is not foveated, and whether enough information is sampled with respect to the task at hand.

### Purpose-Based Approach

For driver attention assessment, the *purpose-based approach* specifically defines which areas a driver must acquire information from to be considered attentive. This requires knowledge about the traffic rules that apply, which, in combination with the situation at hand, indicate where relevant information can be expected given the driver's intended maneuver. To assess the likely reason for a glance (or the absence of a glance), one must also consider the glance history, the infrastructure layout, other road users and traffic regulations. For example, a first glance down the crossing main road is likely meant to check for the presence of traffic. A follow-up glance in the same direction may help determining the available time gap for crossing the road. Here, the speed of the approaching road user may be more important than whether it is a car or a bicyclist. If all areas identified as relevant in the situation have been sampled timely and sufficiently, the driver will be considered attentive according to the purpose-based approach.

The theory of Minimum Required Attention (MiRA; Kircher and Ahlström, 2017) can be used as framework for an *a priori* definition of relevant areas. In Figure 1, one relevant area would be where traffic from the right can be expected (“target area”), regardless of whether traffic is present or not, and the purpose of the glance would thus be to check for traffic. Associated with the target area is a MiRA “zone,” within which the driver must sample information from the target area. This zone is located on the driver's path and its shape is determined by situational circumstances like traffic regulations, line-of-sight, and intended direction of travel. This approach acknowledges that not only the presence but also the absence of other road users is relevant information.

The purpose-based approach explicitly includes the concept of spare capacity (cf. Kujala et al., 2021) by accepting glances to irrelevant areas/targets if all relevant targets are sampled sufficiently. So far, there is no straightforward method to determine when sampling is sufficient, and it appears as if foveal glances are not even necessary in all cases (Wolfe et al., 2017; Vater et al., 2020). Factors like presence, type, trajectory, and speed of other road users are likely to influence sufficiency.

Taking purpose into account leads to a rather different interpretation of the glance in Figure 1. Before crossing the intersection, the driver must check for traffic on the main road. The glance, especially if it is the first glance to the right in this location, is likely intended to check for traffic with right-of-way. With no such traffic present, the salient bicyclist happens to be foveated, even though the bicyclist is not relevant for the driver's upcoming maneuver. A purpose-based interpretation of the glance would be that the driver checked for traffic from the right as required, regardless of the actual target. To determine whether the driver was attentive in the given context, a glance checking for traffic from the left is required too, before the intersection is crossed.

## Discussion

Informative, useful, ET analyses rely on appropriate and reliable gaze data encodings and as we have discussed, these are tools that must be understood in a larger context. The automated data encodings that can be used in direction-based analyses have high objectivity, but they are not always appropriate, because they ignore where in relation to the environment the driver looked and why they looked where they did. For example, coding a glance as “eyes off road” when the driver's gaze is directed to the left (instead of forward) in an upcoming curve is incorrect, because it ignores this context. Opting for a target-based approach, asking what specific object the driver looked at, gives the impression of being more objective and accurate. After all, the driver's gaze either focused on a target or it did not, but the situation is not that simple. A driver's glance over their shoulder may end up being coded as a glance to the guardrail, because that is where foveation happened to occur, even though the intention was to check for overtaking traffic with peripheral vision, which renders the exact location of the fixation irrelevant in the process of acquiring the sought information. This clearly shows the dilemma of having to choose between an almost certainly wrong, but highly reliable coding of the fixated target, and a likely more correct purpose coding, which requires task knowledge and interpretation by the analyst. At least from research in sports there are indications, that in certain situations people fall back on purposely using peripheral vision to save energy and reduce suppression of visual input while the eyes are moving (see also Kredel et al., 2017; Vater et al., 2020).

To ensure reliability in a setting where the analyst's interpretations affect the results, it is important to use data encoding schemes that are well-founded in theoretical models and that suit the research question. In this paper, we use the MiRA theory (Kircher and Ahlström, 2017) to construct our model, although this is not the only possible approach. For example, the safety protocols suggested by Hirsch (1995) could be similarly useful. We do not argue that this approach is the one perfect solution to the problems we have pointed out in ET analyses, merely that it solves some of them. For example, the MiRA theory outlines how relevant areas can be defined, but it does not specify how drivers acquire information from these areas, if foveal vision is required, or if information acquisition via peripheral vision or other sensor modalities is enough. It is important to realize that the chosen theoretical model shapes the coding scheme and dictates what the analyst must infer from observed data. Both aspects have large consequences on the results. As with any new approach, effort must be made to ensure reliability and repeatability. Triangulation with other methods, as well as inter-rater reliability assessments, are good sanity checks for any approach with as many subjective elements as one which includes questions of motivation and reason. That said, being mindful of these limitations, a subjective purpose-based encoding can be more informative than an allegedly objective encoding of glance targets, and regardless of the approach chosen, *a priori* decisions must be made about the data coding scheme.

A key concern underlying our work here, which is unlikely to be alleviated in the near future, is the fact that eye trackers can only measure the gaze direction. They cannot measure information acquired via peripheral vision (Wolfe et al., 2020), spare visual capacity and acquisition of redundant information (Kujala et al., 2021), if fixated targets have been sampled sufficiently (Kircher and Ahlström, 2017), and what is known from past experience (Clark, 2015). In any model determining driver attention, merely knowing where a driver looked is neither sufficient nor adequate. Triangulating data, from multiple methods such as ET (including combinations of the direction-, target-, and purpose based approaches), driving behavior, think aloud (Ericsson and Simon, 1980), visual occlusion (Kujala et al., 2021), and event-related brain potentials (Hopstaken et al., 2016) with theoretical models of peripheral vision and neurocognitive function are likely to be necessary to attain a deeper understanding of driver attention (Kircher and Ahlström, 2018). As an example, by triangulating visual occlusion and ET results, it has been shown that glancing away from the forward roadway for driving purposes is not the same as glancing away for other purposes, and neither is necessarily equivalent to distraction (Kircher et al., 2019). This is, of course, not the only path that could lead to these conclusions, merely one among many.

On the whole, the data that eye trackers provide to driving researchers is immensely valuable, but like any other tool at the researcher's disposal, cannot be viewed as the one arbiter of truth. In this perspective paper, we have laid out ways in which ET data can both be used to better explain the complexities of driver behavior, and how particular ways in which they have been used can be misleading. Future ET research should consider the strengths and weaknesses we have detailed here, with particular attention to why drivers look where they do, what information they acquire foveally and peripherally, how the physical structure of the road environment dictates their behavior, and how their own expertise influences their acquisitive actions. The approach we advocate represents a significant shift in how ET data are used and understood, but it promises to provide key insights into what drivers need to know in a given situation and how they set about gaining the knowledge they require. In essence, the old approaches, unaware as they were of the larger context or motivation behind eye movements, have taken us as far as they can; we propose this complementary and more integrative approach to help researchers understand the complexity of drivers' informational needs and how they satisfy them in the moment.

## Author Contributions

The original ideas for the paper came from KK and CA. All authors contributed equally to the conception of the paper, development of the argument, and writing of the final version.

## Funding

This work was partly funded by the Swedish Strategic Vehicle Research and Innovation Programme (VINNOVA FFI; Grant Number 2019-05834), by Stiftelsen Länsförsäkringsbolagens Forskningsfond, and by the Natural Sciences and Engineering Research Council of Canada (NSERC, RGPIN-2021-02730).

## Conflict of Interest

The authors declare that the research was conducted in the absence of any commercial or financial relationships that could be construed as a potential conflict of interest.

## Publisher's Note

All claims expressed in this article are solely those of the authors and do not necessarily represent those of their affiliated organizations, or those of the publisher, the editors and the reviewers. Any product that may be evaluated in this article, or claim that may be made by its manufacturer, is not guaranteed or endorsed by the publisher.
